# The Advent of Omics Sciences in Clinical Trials of Motor Neuron Diseases

**DOI:** 10.3390/jpm12050758

**Published:** 2022-05-07

**Authors:** Paola Ruffo, Sebastiano Cavallaro, Francesca Luisa Conforti

**Affiliations:** 1 Medical Genetics Laboratory, Department of Pharmacy, Health and Nutritional Sciences, University of Calabria, 87036 Rende, Italy; paolaruffo.bio@gmail.com; 2 Institute for Biomedical Research and Innovation (IRIB), National Research Council (CNR), 95126 Catania, Italy; sebastiano.cavallaro@cnr.it

**Keywords:** clinical trials, omics, personalized medicine, neurodegenerative disease, motor neuron disease, amyotrophic lateral sclerosis, spinal muscular atrophy, genomics, transcriptomics

## Abstract

The “omics revolution” has totally changed the scientific research approach and is contributing to the development of personalized therapies. In motor neuron diseases (MNDs), a set of complex, multifactorial, late-onset and chronic neurodegenerative diseases, the use of multi-omics approaches in clinical trials is providing new opportunities to stratify patients and develop target therapies. To show how omics science is gaining momentum in MNDs, in this work, we review the interventional clinical trials for MNDs based on the application of omics sciences. We analyze a total of 62 clinical trials listed in the ClinicalTrials database where different omics approaches have been applied in an initial phase, for diagnosis or patient selection, or in subsequent stages to cluster subjects, identify molecular signatures or evaluate drugs security or efficacy. The rise of omics sciences in clinical experimentation of MNDs is leading to an upheaval in their diagnosis and therapy that will require significant investments and means to ensure the correct and rapid evolution of personalized medicine.

## 1. Introduction

The Human Genome Project (HGP), devised in the 1980s, began in 1990 and completed in 2003, made it possible to map and sequence the entire human genome. The rapid advancement of knowledge, together with high-throughput sequencing technologies, have laid the foundation of the omics sciences. Several research areas can be classified as omics sciences: genomics examines the genetic composition, transcriptomics investigates gene expression, proteomics focuses on the final protein product, metabolomics analyses metabolites and epigenomics evaluates epigenetic modifications.

In the last years, the application of omics technologies to clinical research has provided a more comprehensive understanding of the pathogenesis of complex diseases and the identification of biomarkers for patient stratification is simplifying the development of target therapies [1]. The field of precision and personalized medicine is, to date, dominated by oncology where the ability to obtain small biopsies allows to screen for somatic mutations and orient a target therapy [2]. In other fields, such as neurology, the same precision medicine approach is more difficult to achieve. In neurological disorders, neural tissue can be biopsied only post-mortem and the identification of molecular traits is more complex [3]. Nonetheless, the application of omics approaches to neurological disorders is deciphering their molecular signatures, allowing the stratification of patients. This approach is also offering the possibility of repurposing disease-modifying drugs or identifying new personalized solutions, evaluating both the useful and adverse effects of drugs.

MNDs represent a group of neurological progressive and degenerative disorders, which selectively affect motor neurons in brain and spinal cord [4]. They include primary lateral sclerosis (PLS), progressive muscular atrophy (PMA), progressive bulbar palsy (PBP), spinal muscular atrophy (SMA), amyotrophic lateral sclerosis (ALS) and spinobulbar muscular atrophy or Kennedy’s disease (SBMA). Although MNDs have quite different aetiologies, the recent explosion in omics sciences is attracting a great deal of clinical research to this field.

To show how omics science is gaining momentum in MNDs, in this manuscript we highlight the rise of the “omics” approach in clinical trials. Following a brief introduction to the MNDs of interest, we review all omics-based clinical trials performed to date to investigate the diagnostic and therapeutic complexity of these rare diseases.

## 2. Clinical Trials in Motor Neuron Diseases

The term “clinical trial” defines a human clinical pharmacological, biomedical or health study that follows predefined protocols. Clinical studies can be observational or interventional: the first tends to demonstrate the possible effects of various risk or protective factors on a group of people by observing events that occur without any intervention by the investigator. Interventional studies are research studies that aim to evaluate a new treatment (therapeutic, diagnostic or surgical), with the aim of evaluating if this represents an improvement compared to what is normally practiced.

**Figure 1:**
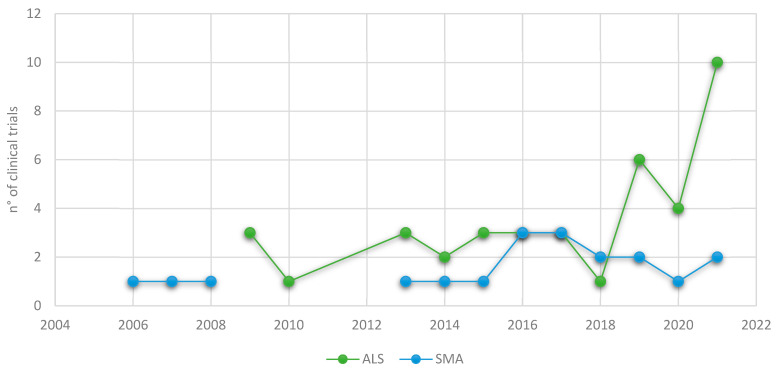
The evolution of interventional clinical trials in ALS and SMA using omics approaches.

Once authorized and before its completion or the publication of results, clinical trials can be searched using dedicated databases, such as ClinicalTrials (available online: https://www.clinicaltrials.gov/, 22 April 2022). To date, 62 omics-based clinical trials for MNDs can be found in this database: 41 (66.1%) are for ALS (Table 1), 20 (32.3%) for SMA (Table 2) and one (1.6%) for Kennedy’s disease. The first trials begun in 2006 and, for ALS, there has been a significant surge in recent years (Figure 1).

Among these clinical trials, 15 (24.2%) studies are currently in Phase I-II, in which the drug’s safety and toxicity as well as the effect produced by the treatment on the pathology under study are assessed; 36 (58.1%) studies are in Phase II-III, in which the drug is tested in a wider number of patients; whereas only one (1.61%) study is in Phase IV, called post-marketing surveillance trial. A genomic approach is used in 23 (37.10%) clinical trials for patient stratification; transcriptomics is used in 16 (25.81%) clinical studies and aims to monitoring therapy, while the remaining 23 (37.10%) trials integrate a multi-omics approach to evaluate the effect of treatments. Finally, grouping all trials according to the purpose for which the omics approach was used, we find that 52 (83.87%) studies aim at drug testing using a high-throughput technology, of which 17 (27.42%) trials aim at patient stratification and 35 (56.45%) at the evaluation of pre- or post-treatment clinical outcomes. The remaining 10 (16.13%) studies use different omics technologies to investigate disease molecular signatures, identify new biomarkers or build repositories.

In the next sections, we will discuss these clinical trials mainly focusing on those completed with published results that permit the evaluation of the usefulness of the omics approaches utilized.

## 3. Amyotrophic Lateral Sclerosis

Amyotrophic Lateral Sclerosis (ALS) is a neurodegenerative disease caused by the progressive loss of motor neurons (MNs) resulting in weakness and paralysis of voluntary muscles. The main clinical feature of ALS is the upper and lower MN involvement. The age of onset is about 60 years, and the incidence is 5 per 100,000 inhabitants [5].

Although important research progress has been made, the etiopathology of ALS is mostly unknown. The mechanisms underlying the development of the disease are multiple, with the involvement of a complex interaction between genetic and molecular characteristics [6]. The major ALS-related genes include superoxide dismutase 1 (*SOD1),* FUSed in sarcoma *(FUS)*, TAR DNA binding protein *(TARDBP)* and chromosome 9 open reading frame 72 (*C9Orf72)* [7,8]. Due to the high complexity of the disease, the diagnosis is made by exclusion and there are no effective drug therapies that can stop or significantly slow down the progression of the disease. To date, the drugs used to slow down the course of the disease are Riluzole [9], which works by reducing excitotoxicity, and Edaravone [10] that decreases oxidative stress.

Neuropathology and medical genetics have led to the discovery that ALS and Frontotemporal Dementia (FTD) are related diseases and form a broad neurodegenerative continuum [11]. Both of these pathologies can be caused by mutations in the same gene that can lead to different clinical pictures [12]. The discovery of hexanucleotide expansion involving the *C9Orf72* gene helped to define a genetic basis to explain the spectrum ALS/FTD [13].

### 3.1. Drug Development

The application of omic sciences has enabled the development of novel approaches to understand the molecular nature of ALS. As described in the following sections, their use has potential clinical implications to stratify patients and identify effective and safe treatments [14,15].

#### 3.1.1. Omics Approach for Patients Stratifications

A genomic approach has been used in different clinical trials to select patients with specific drug-targetable gene mutations. Trial NCT01041222 was the first to use an intrathecally injected antisense oligonucleotide (ASO) designed to inhibit *SOD1* expression in *SOD1*-fALS mutation carriers. The results revealed a successful strategy showing that the drug (ISIS 333611) was well tolerated [16]. A similar genetic stratification was applied in trial NCT00706147, where a genotype-phenotype homogeneous population of *SOD1*-fALS mutation carriers were used to test the safety, tolerability and efficacy of Arimoclomol, a drug promoting the correct folding of proteins. The study demonstrated drug tolerability and safety but did not show therapeutic efficacy [17]. In trial NCT04494256, subjects with *ATXN2* expansion were enrolled to assess the safety, tolerability and pharmacokinetic profile of BIIB105, an ASO designed to bind and degrade the *ATXN2* mRNA.

#### 3.1.2. Omics Approach for Monitoring

A transcriptomic approach has been used to evaluate pre- and post-treatment gene expression changes in ALS patients [18]. In trial NCT04632225, RNA sequencing was used to determine and compare transcription profiles in patients receiving Engensis or placebo. Engensis is a gene therapy based on a plasmid that allows direct expression of the hepatocyte growth factor (HGF) in nerve cells of ALS patients. In trial NCT03359538, the effects of rapamycin in addition to riluzole in ALS patients was assessed by evaluating pathways related to immune response in plasma and cerebrospinal fluid (CSF). A transcriptomic and metabolomic approach was used in trial NCT00875446 to test the safety, pharmacokinetics and pharmacodynamics of the monoclonal antibody GSK1223249 (Ozanezumab), targeting the myelin-associate neurite outgrowth inhibitor (NOGO-A) in ALS patients. The results of this study, conducted on muscle biopsy and plasma by DNA microarray technology, demonstrated the drug is well tolerated although no drug-dependent gene expression patterns in muscle or plasma were identified [19]. The same approach was exploited in trial NCT03456882 to identify pharmacodynamic biomarkers following treatment with RNS60, an electrokinetically altered aqueous fluid.

In several clinical trials, the drug’s impact was determined by comparing the expression of different markers in patients exposed to drugs or placebo (NCT01854294, NCT03800524, NCT04505358, NCT03693781) or observing pre- and post-treatment clinical outcomes (NCT01884571, NCT02525471, NCT02469896, NCT05193994, NCT04788745). Notably, the results of trial NCT01854294 showed that the master regulator peptide GM604 altered plasma expression levels of SOD1, TAU and TDP-43 proteins, slowing down disease progression [20]. In trial NCT01884571, analysis of mRNA expression profiles in blood T-cells was used to assess the effect of immunosuppression and showed no disease-modifying effect following this treatment [21]. By evaluating the effects of Tocilizumab, a monoclonal antibody that inhibits Interleukin-6, trial NCT02469896 demonstrated dysregulation of pro-inflammatory genes in peripheral blood mononuclear cells of sporadic ALS patients [22].

Although discoveries related to the molecular basis of a disease may offer unprecedented opportunities to translate into new drugs, their development requires an enormous amount of time, money, and effort. For this reason, some trials have the objective to repurposing existing drugs to new disease areas [23]. Based on this strategy, the pharmacological properties of Enoxacin were assessed in ALS patients in trial NCT04840823. By acting against bacterial DNA topoisomerase II, this antibacterial agent is used in the treatment of urinary tract infections [24]. Since Enoxacin may regulate the expression of miRNAs, this trial aims to evaluate the ability of this drug in modulating miRNA species in CSF and plasma of ALS patients. Another repurposing drug strategy has been exploited in trial NCT02437110 where the combination of Darunavir, Ritonavir, Dolutegravir, and Tenofovir alafenamide (an antiretroviral therapy approved for human immunodeficiency virus infection) has been tested to suppress the activation of Human Endogenous Retrovirus-K (HERV-K) in ALS patients.

Clinical trial NCT04066244 proposed a multi-omics approach, the NCT04066244 to characterize the safety, tolerability and response of BLZ945, a Colony Stimulating Factor 1 (CSF-1) inhibitor. The outcomes of this trial will be based on genotyping cytochrome P4502C8 (CYP2C8), an enzyme involved in metabolism of xenobiotics.

#### 3.1.3. Multi-Omics Approach for Both Stratification and Monitoring

A genomic, metabolomic and transcriptomic approach has been used to select patients and monitoring therapy. In trials NCT03626012 and NCT04288856, C9Orf72 patients were enrolled to evaluate the safety, tolerability and pharmacokinetics of BIIB078, an ASO targeting C9Orf72 mRNA. A similar approach was applied in trials NCT05163886 and NCT05053035, where C9Orf72 patients were recruited to test the safety, tolerability and biological effect of LAM-002A, (apilimod) an inhibitor of the PIKfyve kinase that works by clearing toxic protein aggregates within lysosomes. The outcome of this trial was to evaluate LAM-002 levels, metabolites and neurofilament light chain (NfL) in plasma and CSF.

Clinical trial NCT04993755 aimed to characterize the safety and tolerability of the reverse transcriptase enzyme inhibitor TPN-101 (censavudine) in C9Orf72 patients with ALS/FTD, ALS or FTD. A similar patient’s classification was used in trial NCT04931862 to test the effect of WVE-004, an ASO designed to mediate the degradation of C9Orf72 mRNA.

A genetic stratification was applied in trial NCT02623699, where SOD1-patients were enrolled to test BIIB067 (tofersen), an ASO designed to degrade *SOD1* mRNA and to prevent protein synthesis. The results of this study confirmed drug safety and revealed drug-dependent *SOD1* expression levels in CSF [25]. The same cohort of patients was used in the extension study NCT03070119 to test long-term treatment with BIIB067. The placebo-controlled trial NCT04856982, proposed to initiate BIIB067 treatment in clinically presymptomatic patients. A similar genetic stratification was applied in trial NCT01083667, where *SOD1* mutation carriers were used to test the antiparasitic drug, Pyrimethamine (Daraprim). The results of this study demonstrated a significant reduction of *SOD1* protein concentrations in CSF [26].

A genomic and metabolomic approach was used in trial NCT04768972 to evaluate the safety, pharmacokinetics and pharmacodynamics of ION363 in FUS-mutation carriers. This drug is an ASO designed to reduce the production of mutated neurotoxic form of *FUS* protein. This successful strategy showed a reduction in CSF of NfL concentration. Using a repurposing drug strategy, *FUS* patients were recruited in trial NCT03707795 to evaluate the effect of Betamethasone by assessing levels of different biomarkers in plasma. A similar strategy was exploited in trial NCT05189106, where the reduction of CSF inflammatory biomarkers was assessed in patients with Alzheimer’s disease and C9Orf72-ALS carriers following treatment with Baricitinib, a JAK inhibitor approved for rheumatoid arthritis and COVID-19 treatment [27].

Clinical trial NCT04220021 used a genomic and transcriptomic approach to evaluate safety and tolerability of Metformin in C9Orf72- patients. Since this drug, approved as a diabetes mellitus remedy, demonstrated beneficial effects through different signalling pathways [28], the trial aim was to evaluate CSF deregulated Repeat-associated non-AUG (RAN) protein levels.

### 3.2. Not Drug Related Clinical Trials

A multi-omics approach has been used in trail NCT02590276 to identify disease signatures in FTD/ALS C9Orf72-carriers [29]. The results implicated dysregulated circulating miRNAs as biomarkers of disease progression [20]. In trial NCT03984708, analysis of metabolic status and associated metabolic pathways in skin biopsy fibroblasts of ALS patients was used to find new therapeutic strategies.

A transcriptomic approach has been used in several clinical studies. In trial NCT01984957, DNA microarray technology was used to identify dysregulated gene expression in muscle biopsies of ALS patients. Combined omics technologies were applied in trial NCT02670226 to identify novel biomarkers in ALS patients by exploration of blood, muscle and satellite cells metabolomes and transcriptomes. A similar approach was applied in clinical trial NCT03851302, where mRNA expression of metabolism genes was assessed in muscle of ALS patients following neuromuscular magnetic stimulation (NMMS).

## 4. Spinal Muscular Atrophy

SMA is a disease characterized by motor neuron degeneration of the spinal cord anterior horns resulting in muscle atrophy and weakness of the trunk and limbs. Based on the age of onset and the severity of symptoms, four different SMA forms can be distinguished: type 1 SMA (SMA1) is the most severe and appears from 6 months of age; type 2 SMA (SMA2) become visible indicatively, between 6 and 18 months of life; type 3 SMA (SMA3) starts after 12 months of age, while type 4 SMA (SMA4) begins in adulthood and is the least severe form. In 95% of cases, the disease is caused by specific mutations in the *SMN1* gene, essential for the function and survival of motor neurons. Patients with SMA have a variable number of second gene copies, *SMN2*, which encodes a shortened form of the SMN protein, with reduced functionality compared to the full SMN protein encoded by the healthy *SMN1* gene. The number of copies of the *SMN2* gene is therefore the basis of the great variability of the disease, with more or less severe forms and a very wide range of symptoms [30]. Due to disease’s rarity, the diagnosis is based on the history and clinical examination of the patients and can be confirmed by appropriate genetic tests. Until recently, treatment of SMA was exclusively symptomatic, based on multidisciplinary approaches aimed at improving the quality of life of patients. Today, however, several specific therapies have been approved for this disease [31].

### 4.1. Drug Development

#### 4.1.1. Omics Approach for Patient Stratifications

A genomic approach for patient stratification has been used in several studies to evaluate the safety, tolerability and pharmacokinetics of a SMN gene-targeted therapy. Trail NCT02122952 was the first to test a replacement gene therapy, based on a self-complementary adeno-associated viral vector-9 (AAV9), to provide a functional copy of the *SMN* gene in SMA1 patients [32]. Encouraging clinical results showed that the drug Onasemnogene Abeparvovec (formerly known as AVXS-101) rapidly improves motor functions in severe cases [32]. In a global study, infants under 6 months of age were enrolled to evaluate this treatment (United States, NCT03306277 [33]; Europe, NCT03461289 [34]; Pacific countries, NCT03837184 [35]). A similar approach has been used in trial NCT03381729 where drug effect was evaluated on infants and children with deletion of 3 copies of *SMN2* and *SMN1* genes. [36].

In trial NCT04576494, adults with genetic SMA type 2 and type 3 were enrolled to study the functional effect of Nusinersen (Spinraza), an ASO designed to allow the *SMN2* gene to produce the full-length and functionally normal protein. This drug has recently been approved by over 40 countries as a treatment for SMA.

#### 4.1.2. Omics Approach for Monitoring

A transcriptomic approach has been used in trial NCT00439569 to evaluate the effect of sodium phenylbutyrate (a histone deacetylase inhibitor) on SMN-mRNA and -protein levels in children with SMA type 2 and type 3. The study was closed prematurely due to poor compliance with drug administration.

#### 4.1.3. Multi-Omics Approach Both for Stratification and Monitoring

A genomic and transcriptomic strategy has been applied in trials NCT03032172, NCT02908685 and NCT02913482 to characterize the safety, tolerability and response of the splice modifier RO7034067 (Risdiplam) in adult, paediatric or infant SMA patients. In these trials, drug effects were assessed by evaluating changes in SMN mRNA and protein levels in the blood following RO7034067 treatment.

A repurposing drug strategy was exploited in trial NCT00485511 where the efficacy and safety of Hydroxyurea (HU) was tested in children with *SMN1*-*SMN2* mutations. By reducing the need for blood transfusions in patients with sickle cell anaemia, this drug is used for the treatment of chronic myeloid leukaemia as well as in the prevention of painful episodes [37].

### 4.2. Not Drug Related Clinical Trials

A genomic approach has been applied in trial NCT02550691 to identify specific cis duplication biomarker of *SMN1* in SMA families and, in trial NCT04833348, to quantify motor function in infants treated with innovative therapies (gene therapy or pharmacogenomics).

## 5. Spinal Bulbar Muscular Atrophy

SBMA, or Kennedy disease, is a genetic late onset MND characterized by slowly progressive weakness of the bulbar muscles and limbs with fasciculations, muscle atrophy and gynecomastia [38]. The disorder is clinically similar to, but genetically distinct from classical forms of autosomal SMA. SBMA is caused by a CAG repeat expansion in the first exon of the androgen receptor (AR) gene, on the X chromosome, encoding for a poly-glutamine (polyQ) tract. A repeat number higher than 38 is considered pathogenic. Usually it only affects men, although female carriers may have a mild form of the disease [39]. To date, no disease-modifying treatments for SBMA are available [39].

A genomic approach has been used in clinical trial NCT00303446 to evaluate the pharmacological properties of dutasteride in SBMA patients. By acting as a 5-alpha-reductase I and II inhibitor, this treatment is used against benign prostatic hyperplasia [40]. The results of this study demonstrated drug tolerability but did not show beneficial effect on muscle strength [41].

## 6. Conclusions

Omics sciences are quickly modifying clinical investigations of MNDs to achieve a more comprehensive view of underlying molecular pathways and allow patients stratification into diagnostic, prognostic and therapeutic subgroups. Today we are at a critical point in the development of personalized medicines for MNDs. The landscape of clinical trials using omics approaches and their surge in recent years are expected to produce an important transformation of clinical management of MNDs. The omics-based development of newly launched therapies is speeding solutions to the clinic and have a profound impact in both diagnostics and therapy. However, the road to translate these solutions to the clinic can be challenging. There is still a wide gap between discovery research and clinical practice. Bridging this gap and allowing an effective and sustainable implementation of personalized medicine will require new investments, technologies as well as the training of health professionals. The lack of these may result in inequities in the access or delivering of person-centred care.

## Figures and Tables

**Table 1:** List of clinical trials carried out on ALS using different omics approaches.

Amyotrophic Lateral Sclerosis										
Clinical Trials Identifier	Date		Phase	Status	Treatment		Approach	Title	State	Phase
	Start	Completion			Name	Drug				
**NCT01041222**	January 2010	January 2012	Completed	fALS diagnosis: *SOD1* gene mutation carries	ISIS 333611	ASO designed to inhibit *SOD1* expression	Genomics	A Phase 1, Double-Blind, Placebo-Controlled, Dose-Escalation Study of the Safety, Tolerability, and Pharmacokinetics of ISIS 333,611 Administered Intrathecally to Patients with Familial Amyotrophic Lateral Sclerosis Due to Superoxide Dismutase 1 Gene Mutations	United States	I
**NCT00706147**	January 2009	December 2014	Completed	*SOD1* genetic mutation	Arimoclomol	HSP response inductor	Genomics	Phase II/III Randomized, Placebo-Controlled Trial of Arimoclomol in *SOD1* Positive Familial Amyotrophic Lateral Sclerosis (ALS).	United States	II-III
**NCT04494256**	28 September 2020		Recruiting	Genetic diagnosis (*SOD1*, *FUS*, *ATXN2*)	BIIB105	ASO is designed to bind *ATXN2* mRNA and mediate its degradation	Genomics	A Phase 1 Multiple-Ascending-Dose Study to Assess the Safety, Tolerability, and Pharmacokinetics of BIIB105 Administered Intrathecally to Adults with Amyotrophic Lateral Sclerosis with or without Poly-CAG Expansion in the Ataxin-2 Gene.	United States	I
**NCT04632225**	9 February 2021		Active, not recruiting	ALS diagnosis according to the El Escorial Criteria	Engensis	Gene therapy using plasmid to deliver the *HGF* gene directly to nerve cells	Transcriptomics	A Phase 2a, Double-Blind, Randomized, Placebo-Controlled, Multicenter Study to Assess the Safety of Engensis in Participants with Amyotrophic Lateral Sclerosis	United States	II
**NCT03359538**	19 September 2017		Active, not recruiting	ALS diagnosis according to the El Escorial Criteria	Rapamycin—Sirolimus	Immunomodulatory effects and improves protein degradation	Transcriptomics	Rapamycin (Sirolimus) Treatment for Amyotrophic Lateral Sclerosis	Italy	II
**NCT00875446**	13 May 2009	9 September 2011	Completed	ALS diagnosis according to the Gold Coast Criteria	GSK1223249- Ozanezumab	Monoclonal antibody targeting NOGO-A protein	Transcriptomics and metabolomics	A Single and Repeat Dose Escalation Study of the Safety, Pharmacokinetics and Pharmacodynamics of GSK1223249 in ALS Patients	United States	I
**NCT03456882**	18 November 2016	23 November 2020	Completed	ALS diagnosis according to the El Escorial Criteria	RNS60	Saline solution with charged oxygenated nanobubbles	Metabolomics and transcriptomics	The Effect of RNS60 on ALS Biomarkers.	Italy	II
**NCT01854294**	August 2013	April 2014	Completed	ALS diagnosis according to the El Escorial Criteria	GM604	Peptide	Transcriptomics	GM604 Phase 2A Randomized Double-blind Placebo Controlled Pilot Trial in Amyotrophic Lateral Disease (ALS)	United States	II
**NCT03800524**	22 February 2019		Recruiting	ALS diagnosis according to the El Escorial Criteria	Tauroursodeoxycholic	Antiapoptotic and ER stress response damping effects	Transcriptomics	Safety and Efficacy of Tauroursodeoxycholic (TUDCA) as add-on Treatment in Patients Affected by Amyotrophic Lateral Sclerosis (ALS)	Belgium, France and others	III
**NCT04505358**	30 December 2021		Not yet recruiting	ALS diagnosis according to the El Escorial Criteria	PU-AD— Icapamespib	Brain permeable Hsp90 protein inhibitor	Genomics and transcriptome	A Randomized, Double-blind, Placebo-controlled Pilot Study to Evaluate the Biological Activity, Safety, and Pharmacokinetics of PU-AD in Subjects with Amyotrophic Lateral Sclerosis (ALS)		II
**NCT03693781**	10 April 2019		Active, not recruiting	ALS diagnosis according to the El Escorial Criteria	Colchicine		Transcriptomics	Colchicine for Amyotrophic Lateral Sclerosis: A Phase II, Randomized, Double Blind, Placebo Controlled, Multicenter Clinical Trial	Italy	II
**NCT01884571**	October 2013	January 2016	Completed	ALS diagnosis according to the El Escorial Criteria	Basiliximab, Methylprednisolone, Prednisone, Tacrolimus, Mycophenolate mofetil	Immunosuppression treatment	Transcriptomics	A Novel Immunosuppression Intervention for the Treatment of Amyotrophic Lateral Sclerosis (ALS)	United States	II
**NCT02525471**	October 2015	21 June 2017	Completed	ALS diagnosis according to the El Escorial Criteria	RNS60	Saline solution with charged oxygenated nanobubbles	Transcriptomics	A Pilot Study of RNS60 in Amyotrophic Lateral Sclerosis (ALS)	United States	I
**NCT02469896**	November 2015	11 July 2018	Completed	ALS diagnosis according to the El Escorial Criteria	Tocilizumab	ASO designed to inhibit interleukin 6 (IL-6)	Transcriptomics	A Phase 2 Randomized, Placebo Controlled Trial of Tocilizumab in ALS Subjects	United States	II
**NCT05193994**	18 January 2022		Not yet recruiting	ALS diagnosis according to the Gold Coast Criteria	Triumeq	Combined treatment of: dolutegravir, abacavir, lamivudine	Genomics and transcriptomics	Randomised Double-Blind Placebo-Controlled Phase 3 Trial of Triumeq in Amyotrophic Lateral Sclerosis	Australia	III
**NCT04788745**	29 June 2021		Recruiting	ALS diagnosis according to the El Escorial Criteria	Trimetazidine Dihydrochloride		Transcriptomics	Targeting Metabolic Flexibility in ALS (MetFlex); Safety and Tolerability of Trimetazidine for the Treatment of ALS	Australia, United Kindom and others	II
**NCT04840823**	26 March 2021		Recruiting	ALS diagnosis according to the El Escorial Criteria	Enoxacin	Quinolone/fluoroquinolone antibiotic	Transcriptomics	A Randomized, Double-blind, Parallel Group, Single Centre, Phase 1b/2 Study to Assess the Safety, Tolerability, Pharmacokinetics and Pharmacodynamics of Three Orally Administered Doses of Enoxacin (200 mg Twice Daily, 400 mg Twice Daily and 600 mg Twice Daily) in Adults with Amyotrophic Lateral Sclerosis	Canada	I-II
**NCT02437110**	1 April 2019		Recruiting	ALS diagnosis according to the El Escorial Criteria	Darunavir, Ritonavir, Dolutegravir, Tenofovir alafenamide (TAF)	Darunavir and Ritonavir = protease inhibitor; Dolutegravir = integrase inhibitor; TAF = nucleoside reverse transcriptase inhibitor	Transcriptomics	HERV-K Suppression Using Antiretroviral Therapy in Volunteers with Amyotrophic Lateral Sclerosis (ALS)	United States	I
**NCT04066244**	30 December 2019		Recruiting	ALS diagnosis according to the El Escorial Criteria	BLZ945	CSF-1 Inhibitor	Genomics Metabolomics and transcriptomics	An Open-label, Adaptive Design Study in Patients with Amyotrophic Lateral Sclerosis (ALS) to Characterize Safety, Tolerability and Brain Microglia Response, as Measured by TSPO Binding, Following Multiple Doses of BLZ945 Using Positron Emission Tomography (PET) With the Radioligand [11C]-PBR28	United States	II
**NCT03626012**	10 September 2018	17 November 2021	Completed	*C9Orf72* genetic mutation	BIIB078	ASO designed to target C9Orf72 mRNA	Genomics and metabolomics	A Phase 1 Multiple-Ascending-Dose Study to Assess the Safety, Tolerability, and Pharmacokinetics of BIIB078 Administered Intrathecally to Adults with *C9ORF72*-Associated Amyotrophic Lateral Sclerosis	United States, Belgium and others	I
**NCT04288856**	28 April 2020		Active, not recruiting	*C9Orf72* genetic mutation	BIIB078	ASO designed to target C9Orf72 mRNA	Genomics and metabolomics	An Extension Study to Assess the Long-Term Safety, Tolerability, Pharmacokinetics, and Effect on Disease Progression of BIIB078 Administered to Previously Treated Adults with *C9ORF72*-Associated Amyotrophic Lateral Sclerosis	United States	I
**NCT05163886**	23 December 2021		Recruiting	*C9Orf72* genetic mutation	LAM-002A	PIKfyve kinase inhibitor that activates the transcription factor EB (TFEB)	Genomics and transcriptome	A Phase IIa Trial to Evaluate the Safety, Tolerability, and Biological Activity of LAM-002A (Apilimod Dimesylate Capsules) in *C9ORF72*-Associated Amyotrophic Lateral Sclerosis	United States	II
**NCT05053035**	2 September 2021		Recruiting	*C9Orf72* genetic mutation	LAM-002A	PIKfyve kinase inhibitor that activates the transcription factor EB (TFEB)	Genomics and transcriptome	A Phase 2, Multicenter, Randomized, Double-Blind, Placebo-Controlled Study to Evaluate the Safety, Tolerability, Pharmacokinetics, and Pharmacodynamics of AL001 in *C9orf72*-Associated Amyotrophic Lateral Sclerosis	United States	II
**NCT04993755**	1 October 2021		Recruiting	*C9Orf72 * genetic mutation	TPN-101, censavudine	Inhibitor of the reverse transcriptase enzyme	Genomics and metabolomics	A Phase 2a Study of TPN-101 in Patients with Amyotrophic Lateral Sclerosis (ALS) and/or Frontotemporal Dementia (FTD) Associated with Hexanucleotide Repeat Expansion in the *C9Orf72* Gene (*C9ORF72* ALS/FTD)	United States	II
**NCT04931862**	28 June 2021		Recruiting	*C9Orf72* genetic mutation	WVE-004	ASO is designed to mediate the degradation of C9ORF72 mRNAs	Genomics	A Multicenter, Randomized, Double-blind, Placebo-controlled, Phase 1b/2a Study of WVE-004 Administered Intrathecally to Patients with *C9orf72*-associated Amyotrophic Lateral Sclerosis (ALS) or Frontotemporal Dementia (FTD)	Australia	I-II
**NCT02623699**	20 January 2016	16 July 2021	Completed	*SOD1* genetic mutation	BIIB067—Tofersen	ASO designed to degrade *SOD1* mRNA to prevent protein synthesis and reduce levels of harmful proteins.	Genomics and metabolomics	A Study to Evaluate the Efficacy, Safety, Tolerability, Pharmacokinetics, and Pharmacodynamics of BIIB067 Administered to Adult Subjects with Amyotrophic Lateral Sclerosis and Confirmed Superoxide Dismutase 1 Mutation	United States	III
**NCT03070119**	8 March 2017		Active, not recruiting	*SOD1* genetic mutation	BIIB067—Tofersen	ASO designed to degrade *SOD1* mRNA to prevent protein synthesis and reduce levels of harmful proteins.	Genomics	An Extension Study to Assess the Long-Term Safety, Tolerability, Pharmacokinetics, and Effect on Disease Progression of BIIB067 Administered to Previously Treated Adults with Amyotrophic Lateral Sclerosis Caused by Superoxide Dismutase 1 Mutation	United States	III
**NCT04856982**	17 May 2021		Recruiting	*SOD1* genetic mutation	BIIB067—Tofersen	ASO designed to degrade *SOD1* mRNA to prevent protein synthesis and reduce levels of harmful proteins.	Genomics and metabolomics	A Phase 3 Randomized, Placebo-Controlled Trial with a Longitudinal Natural History Run-In and Open-Label Extension to Evaluate BIIB067 Initiated in Clinically Presymptomatic Adults with a Confirmed Superoxide Dismutase 1 Mutation	United States	III
**NCT01083667**	November 2009	December 2014	Completed	*SOD1* genetic mutation	Pyrimethamine – Daraprim		Genomics and metabolomics	Phase I/II Study of *SOD1* Inhibition by Pyrimethamine in Familial ALS	United States, Germany and others	I-II
**NCT04768972**	14 June 2021		Recruiting	*FUS* genetic mutation	ION363—Jaci*FUS* en	ASO designed to reduce the production of a mutated neurotoxic form of the FUS protein	Genomics and metabolomics	A Phase 1–3 Study to Evaluate the Efficacy, Safety, Pharmacokinetics and Pharmacodynamics of Intrathecally Administered ION363 in Amyotrophic Lateral Sclerosis Patients with FUS ed in Sarcoma Mutations (*FUS* -ALS)	United States	III
**NCT03707795**	21 August 2017	10 January 2019	Completed	*FUS* genetic mutation and fALS diagnosis	Betamethasone	Corticosteroid, reducing inflammation and changing the body’s immune response	Genomics and metabolomics	Treatment of *FUS* -Related ALS With Betamethasone—The TRANSLATE Study	United States	Early I
**NCT05189106**	1 February 2022		Not yet recruiting	ALS diagnosis according to the El Escorial Criteria	Baricitinib— Olumiant	Immunosuppressant—JAK inhibitors	Genomics, proteomics and metabolomics	Neurodegenerative Alzheimer’s Disease and Amyotrophic Lateral Sclerosis (NADALS) Basket Proof of Concept Trial Including Asymptomatic Individuals Using Baricitinib	United States	I-II
**NCT04220021**	10 January 2020		Recruiting	*C9Orf72* genetic mutation	Metformin		Genomics and metabolomics	A Single-Center, Open Label Study to Assess the Safety and Tolerability of Metformin in Subjects with *C9orf72* Amyotrophic Lateral Sclerosis Over 24 Weeks of Treatment	United States	II
**NCT02590276**	8 October 2015	27 October 2020	Completed	*C9Orf72* genetic mutation			Genomics, Metabolomics and transcriptomics	Predict to Prevent Frontotemporal Lobar Degeneration and Amyotrophic Lateral Sclerosis	France	Not Applicable
**NCT03984708**	27 January 2020		Recruiting	ALS diagnosis according to the El Escorial Criteria			Metabolomics, lipidpomics and transcriptomics	New Therapeutic Strategy in ALS Based on Metabolic Status and Associated Metabolic Pathways.	France	Not Applicable
**NCT01984957**	January 2013	January 2015	Completed	ALS diagnosis according to the El Escorial Criteria			Transcriptomics	Differential Study of Muscle Transcriptome in Patients with Neuromuscular Disease and Control Subjects	France	Not Applicable
**NCT02670226**	29 March 2016	9 December 2019	Completed	ALS diagnosis according to the El Escorial Criteria			Metabolomics and transcriptomics	Metabolomics and Transcriptomics Approaches to Identify Muscular Biomarkers in Amyotrophic Lateral Sclerosis	France	Not Applicable
**NCT03851302**	28 October 2019		Recruiting	ALS diagnosis according to the El Escorial Criteria			Transcriptomics	Effects of Remote Ischemic Conditioning on Hand Use in Individuals with Spinal Cord Injury and Amyotrophic Lateral Sclerosis: A Preliminary Study	United States	Not Applicable
**NCT03618966**	1 November 2014	1 May 2016	Completed	ALS diagnosis according to the El Escorial Criteria			Transcriptomics	Neuromuscular Magnetic Stimulation Counteracts Muscle Decline in ALS Patients		II
**NCT03367650**	13 May 2014		Recruiting	ALS diagnosis according to the El Escorial Criteria			Genomics	Epidemiology and Genetics of the Amyotrophic Lateral Sclerosis in the French West Indies	France	Not Applicable
**NCT03573466**	10 April 2019		Active, not recruiting	*SOD1*- *C9Orf72* genetic mutation			Genomics	Presymptomatic Neuromuscular Junction Defects and Compensatory Mechanisms in Amyotrophic Lateral Sclerosis (ALS)	France	Not Applicable

The search for clinical trials in the ClinicalTrials.gov database was carried out using “Amyotrophic Lateral Sclerosis” and “mutation”, “mutational”, “gene expression”, “genotype”, “gene”, “transcriptome”, “transcriptomics”, “*C9Orf72*”, “*SOD1*”, “*FUS*”, “*TARDBP*”, “DNA”, “RNA”, “sequencing” as keywords. The names of genes are in italics.

**Table 2:** List of clinical trials carried out on SMA and SBMA using different omics approaches.

Spinal Muscular Atrophy and Spinal-Bulbar Muscular Atrophy										
Clinical Trials Identifier	Date		Phase	Status	Treatment		Approach	Title	State	Phase
	Start	Completion			Name	Drug				
**NCT02122952**	5 May 2014	15 December 2017	Completed	*SMN1*–*SMN2* genetic diagnosis	AVXS-101—Onasemnogene Abeparvovec	*SMN* gene therapy	Genomics	Phase I Gene Transfer Clinical Trial for Spinal Muscular Atrophy Type 1 Delivering AVXS-101	United States	I
**NCT03306277**	24 October 2017	12 November 2019	Completed	*SMN1*–*SMN2* genetic diagnosis	AVXS-101—Onasemnogene Abeparvovec	*SMN* gene therapy	Genomics	Phase 3, Open-Label, Single-Arm, Single-Dose Gene Replacement Therapy Clinical Trial for Patients with Spinal Muscular Atrophy Type 1 With One or Two *SMN2* Copies Delivering AVXS-101 by Intravenous In*FUS* ion	United States	III
**NCT03461289**	16 August 2018	11 September 2020	Completed	*SMN1*–*SMN2* genetic diagnosis	AVXS-101—Onasemnogene Abeparvovec	*SMN* gene therapy	Genomics	Phase 3, Open-Label, Single-Arm, Single-Dose Gene Replacement Therapy Clinical Trial for Patients with Spinal Muscular Atrophy Type 1 With One or Two *SMN2* Copies Delivering AVXS-101 by Intravenous In*FUS* ion	Belgium, France and others	III
**NCT03837184**	31 May 2019	29 June 2021	Completed	*SMN1*–*SMN2* genetic diagnosis	AVXS-101—Onasemnogene Abeparvovec	*SMN* gene therapy	Genomics	Phase 3, Open-Label, Single-Arm, Single-Dose Gene Replacement Therapy Clinical Trial for Patients with Spinal Muscular Atrophy Type 1 With One or Two *SMN2* Copies Delivering AVXS-101 by Intravenous In*FUS* ion	Japan, Korea and Taiwan	III
**NCT03381729**	14 December 2017	18 November 2021	Completed	*SMN2* genetic diagnosis	AVXS-101—Onasemnogene Abeparvovec	*SMN* gene therapy	Genomics	Phase I, Open-Label, Dose Comparison Study of AVXS-101 for Sitting but Non-ambulatory Patients with Spinal Muscular Atrophy	United States	I
**NCT03505099**	2 April 2018	15 June 2021	Completed	*SMN2* genetic diagnosis	AVXS-101—Onasemnogene Abeparvovec	*SMN* gene therapy	Genomics	A Global Study of a Single, One-Time Dose of AVXS-101 Delivered to Infants with Genetically Diagnosed and Pre-symptomatic Spinal Muscular Atrophy with Multiple Copies of *SMN2*	United States	III
**NCT04042025**	10 February 2020		Enrolling by invitation	SMA clinical and genetic diagnosis	AVXS-101—Onasemnogene Abeparvovec	*SMN* gene therapy	Genomics	A Long-term Follow-up Study of Patients in the Clinical Trials for Spinal Muscular Atrophy Receiving AVXS-101	United States	IV
**NCT02628743**	20 January 2016	18 December 2018	Completed	*SMN2* genetic diagnosis	AVXS-101—Onasemnogene Abeparvovec	*SMN* gene therapy	Genomics	Multicenter, Open-Label, Single-Arm Study to Evaluate Long-Term Safety, Tolerability, and Effectiveness of 10 mg/kg BID Olesoxime in Patients with Spinal Muscular Atrophy	Belgium, France and others.	II
**NCT04576494**	24 January 2022		Recruiting	SMA genetic diagnosis	Nusinersen—Spinraza	ASO designed to allow the *SMN2* gene to produce the full-length protein that can function normally	Genomics	Study of the Functional Effects of Nusinersen in 5q-spinal Muscular Amyotrophy Adults (SMA Type 2 or 3 Forms): a Multicenter Single-case Experimental Design in Multiple Baselines Across Subjects, Randomized, Single-blinded Evaluation	France	Not Applicable
**NCT04851873**	8 September 2021		Recruiting	*SMN1*–*SMN2* genetic diagnosis	OAV101 (AVXS-101)	*SMN* gene therapy	Genomics	A Phase IIIb, Open-label, Single-arm, Single-dose, Multicenter Study to Evaluate the Safety, Tolerability and Efficacy of Gene Replacement Therapy with Intravenous OAV101 (AVXS-101) in Pediatric Patients With Spinal Muscular Atrophy (SMA)	Australia	III
**NCT05089656**	2 February 2022		Recruiting	*SMN1*–*SMN2* genetic diagnosis	OAV101 (AVXS-101)	*SMN* gene therapy	Genomics	A Randomized, Sham-controlled, Double-blind Study to Evaluate the Efficacy and Safety of Intrathecal OAV101 in Patients Type 2 Spinal Muscular Atrophy (SMA) Who Are ≥ 2 to < 18 Years of Age, Treatment Naive, Sitting, and Never Ambulatory	United States	III
**NCT03779334**	7 August 2019		Active, not recruiting	*SMN2* genetic diagnosis	RO7034067- Risdiplam	A splice modifier of the pre-mRNA of the *SMN2* gene	Genomics	An Open-Label Study of Risdiplam in Infants with Genetically Diagnosed and Presymptomatic Spinal Muscular Atrophy	United States	II
**NCT01671384**	August 2013		Unknown	*SMN1* genetic diagnosis	Valproate and levocarnitine	Valproic acid (VPA) = a histone deacetylase inhibitor (HDAC)	Genomics	Randomized Placebo Controlled Trial of Valproate and Levocarnitine in Children with Spinal Muscular Atrophy Aged 2–15 Years	India	III
**NCT00439569**	January 2008	August 2008	Terminated	SMA clinical diagnosis	Sodium phenylbutyrate	Histone deacetylase inhibitor and a chemical chaperone	Transcriptomics	Phase I/IIa Clinical Trial of Sodium Phenylbutyrate in Pediatric Subjects with Type II/III Spinal Muscular Atrophy	United States	I-II
**NCT03032172**	3 March 2017		Active, not recruiting	*SMN2* genetic diagnosis	RO7034067- Risdiplam	A splice modifier of the pre-mRNA of the *SMN2* gene	Transcriptomics	An Open-Label Study to Investigate the Safety, Tolerability, and Pharmacokinetics/Pharmacodynamics of Risdiplam (RO7034067) in Adult and Pediatric Patients with Spinal Muscular Atrophy	United States	II
**NCT02908685**	20 October 2016		Active, not recruiting	SMA genetic diagnosis	RO7034067- Risdiplam	A splice modifier of the pre-mRNA of the *SMN2* gene	Genomics and transcriptomics	A Two Part Seamless, Multi-Center Randomized, Placebo-Controlled, Double-Blind Study to Investigate the Safety, Tolerability, Pharmacokinetics, Pharmacodynamics and Efficacy of Risdiplam (RO7034067) in Type 2 and 3 Spinal Muscular Atrophy Patients	United States	II-III
**NCT02913482**	23 December 2016		Active, not recruiting	SMA genetic diagnosis	RO7034067- Risdiplam	A splice modifier of the pre-mRNA of the *SMN2* gene	Genomics and transcriptomics	A Two Part Seamless, Open-label, Multicenter Study to Investigate the Safety, Tolerability, Pharmacokinetics, Pharmacodynamics and Efficacy of Risdiplam (RO7034067) in Infants with Type 1 Spinal Muscular Atrophy	United States	II-III
**NCT00485511**	June 2007	June 2009	Completed	*SMN1*–*SMN2* genetic diagnosis	Hydroxyurea	*SMN2* transcription pattern modified	Genomics and transcriptomics	A Randomized, Double-Blind, Placebo-Controlled Trial of Hydroxyurea in Spinal Muscular Atrophy	Taiwan	II-III
**NCT02550691**	15 December 2015	4 July 2016	Terminated	*SMN1*–*SMN2* genetic diagnosis			Genomics	Identification of a Biomarker Associated with Cis-duplication of the *SMN1* Gene Aiming at Improving the Genetic Counseling in Spinal Muscular Atrophy Families	France	Not Applicable
**NCT04833348**	August 2021		Not yet recruiting	*SMN1* genetic diagnosis			Genomics	Quantification of Motor Function in Infants with Spinal Muscular Atrophy Treated with Innovative Therapies, IMUSMA Project	France	Not Applicable
**NCT00303446**	March 2006	December 2009	Completed	SBMA genetically confirmed	Dutasteride	Inhibitor of 5-alpha-reductase I and II	Genomics	Dutasteride to Treat Spinal and Bulbar Muscular Atrophy (SBMA)	United States	II

The search for clinical trials in the ClinicalTrials.gov database was carried out using the “Spinal Muscular Atrophy” or “Spinal-Bulbar Muscular Atrophy” and “mutation”, “mutational”, “gene expression”, “genotype”, “gene”, “transcriptome”, “transcriptomics”, “*C9Orf72*”, “*SOD1*”, “*FUS*”, “*TARDBP*”, “DNA”, “RNA”, “sequencing” as keywords. The names of genes are in italics.

## Data Availability

Not applicable.
